# Draft Genome Sequences of Clinical and Nonclinical Isolates of *Klebsiella* spp. Exhibiting Nonheritable Tolerance toward Antimicrobial Compounds

**DOI:** 10.1128/genomeA.01217-17

**Published:** 2017-11-09

**Authors:** Shashank Patole, Mitali Mishra, Harapriya Mohapatra

**Affiliations:** School of the Biological Sciences, National Institute of Science Education and Research, HBNI, Bhubaneswar, Odisha, India

## Abstract

A clinical isolate and a nonclinical isolate of *Klebsiella pneumoniae* were found to exhibit nonheritable tolerance in response to antimicrobial compounds. The draft genome sequences of both isolates are presented here.

## GENOME ANNOUNCEMENT

*Klebsiella pneumoniae* is found in natural habitats, such as soil and water and on vegetation, and is known to cause a variety of nosocomial infections in immunocompromised individuals, such as wound infections, urinary tract infections, and respiratory tract infections (1, 2). Further, the past decade has seen a drastic rise in community-acquired *Klebsiella pneumoniae* infections (3–6). Previously, we studied the association between multiple-antibiotic resistance and virulence in environmental bacterial isolates, including those belonging to *Klebsiella* (7). We observed the environmental *K. quasipneumoniae* isolate DL5.4 (GenBank accession number JQ912548) to exhibit nonheritable tolerance toward antimicrobial compounds. Simultaneously, we observed a persistence phenomenon in the wound infection isolate KpIMS38, which was obtained from a tertiary-care hospital in Bhubaneswar, India. Further, we noticed that the isolate KpIMS38 harbored a plasmid. This study reports whole-genome sequencing of these two isolates that will enable genomic comparisons specifically with respect to their sources of isolation and persister-forming ability.

Genomic DNA from both the isolates and plasmid DNA from KpIMS38 were extracted using the Gentra Puregene Yeast/Bact. kit and the QIAprep spin miniprep kit (Qiagen GmbH), respectively, according to the manufacturer’s instructions. Whole-genome sequencing was carried out at a laboratory of Thermo Fisher Scientific, Gurgaon, India. Briefly, libraries were prepared for individual genomes and the plasmid using the workflow delineated by the Ion Xpress Plus fragment library kit (Thermo Fisher Scientific, USA), amplified using the Ion OneTouch 2 system (Thermo Fisher Scientific), and sequenced using the Ion S5 system (Thermo Fisher Scientific). A total of 597,006,766, 400,449,596, and 18,357,749 bases in the form of 1,700,049, 1,151,033, and 60,120 reads were obtained, with average read lengths of 351, 348, and 305 bp for KpIMS38, DL5.4, and the plasmid, respectively. These were assembled using the SPAdes algorithm version 3.1.0 (8) into 169, 170, and 71 contigs, with average sizes of 72,636, 84,693, and 398 bases for KpIMS38, DL5.4, and the plasmid DNA, respectively.

The Rapid Annotations using Subsystems Technology (RAST) server (9, 10) was used to annotate the genomes of KpIMS38 and DL5.4, which were 5,255,239 (inclusive of the 13,170-bp plasmid) and 5,134,131 bp in size, with G+C contents of 57.2% and 58.2%, respectively. Isolates KpIMS38 and DL5.4 contained 4,320 and 4,295 protein-coding genes with assigned functions, while 1,131 and 910 genes were annotated as coding for hypothetical proteins. Furthermore, 92 and 97 genes were found to code for RNA in KpIMS38 and DL5.4, respectively. The sequences have also been submitted to the Prokaryotic Genome Annotation Pipeline (PGAP) (11). The findings from this study will facilitate further analysis of gene function association studies with respect to the phenomenon of persistence.

### Accession number(s).

This whole-genome shotgun project has been deposited in DDBJ/EMBL/GenBank under the accession numbers NQMT00000000 and NMPY00000000 for the isolate KpIMS38 with its plasmid sequence and the isolate DL5.4, respectively. The versions described in this paper are the first versions for both and are publicly available.
